# Extraction of Cellulose Nanofibers via Eco-friendly Supercritical Carbon Dioxide Treatment Followed by Mild Acid Hydrolysis and the Fabrication of Cellulose Nanopapers

**DOI:** 10.3390/polym11111813

**Published:** 2019-11-05

**Authors:** M. S. Nurul Atiqah, Deepu A. Gopakumar, Owolabi F. A. T., Yasir Beeran Pottathara, Samsul Rizal, N. A. Sri Aprilia, D. Hermawan, M. T. Paridah, Sabu Thomas, Abdul Khalil H. P. S.

**Affiliations:** 1 School of Industrial Technology, Universiti Sains Malaysia, Penang 11800, Malaysia; nurulatiqah.msaad@gmail.com (M.S.N.A.); deepu1789@gmail.com (D.A.G.);; 2 Federal Institute of Industrial Research Oshodi, Ikeja Lagos PMB 21023, Nigeria; 3 Faculty of Mechanical Engineering, University of Maribor, 2000 Maribor, Slovenia; ptyasirbeeran@gmail.com; 4 Department of Mechanical Engineering, Universitas Syiah Kuala, Banda Aceh 23111, Indonesia; samsul_r@yahoo.com; 5 Department of Chemical Engineering, Universitas Syiah Kuala, Banda Aceh 23111, Indonesia; sriaprilia@unsyiah.ac.id; 6 Department of Forest Product, Faculty of Forestry, Kampus IPB, Darmaga, Bogor Agricultural University, Bogor 16001, West Java, Indonesia; mr.dede.hermawan@gmail.com; 7 Institute of Tropical Forestry and Forest Products (INTROP), Universiti Putra Malaysia, Serdang, Selangor 43400, Malaysia; 8 School of Chemical Sciences, Mahatma Gandhi University, Kottayam, Kerala 686560, India; sabuthomas@mgu.ac.in

**Keywords:** kenaf fiber, total chlorine free bleaching, supercritical carbon dioxide, cellulose nanofibers, cellulose nanopaper

## Abstract

The conventional isolation of cellulose nanofibers (CNFs) process involves high energy input which leads to compromising the pulp fiber’s physical and chemical properties, in addition to the issue of elemental chlorine-based bleaching, which is associated with serious environmental issues. This study investigates the characteristic functional properties of CNFs extracted via total chlorine-free (TCF) bleached kenaf fiber followed by an eco-friendly supercritical carbon dioxide (SC-CO_2_) treatment process. The Fourier transmission infra-red FTIR spectra result gave remarkable effective delignification of the kenaf fiber as the treatment progressed. TEM images showed that the extracted CNFs have a diameter in the range of 10–15 nm and length of up to several micrometers, and thereby proved that the supercritical carbon dioxide pretreatment followed by mild acid hydrolysis is an efficient technique to extract CNFs from the plant biomass. XRD analysis revealed that crystallinity of the fiber was enhanced after each treatment and the obtained crystallinity index of the raw fiber, alkali treated fiber, bleached fiber, and cellulose nanofiber were 33.2%, 54.6%, 88.4%, and 92.8% respectively. SEM images showed that amorphous portions like hemicellulose and lignin were removed completely after the alkali and bleaching treatment, respectively. Moreover, we fabricated a series of cellulose nanopapers using the extracted CNFs suspension via a simple vacuum filtration technique. The fabricated cellulose nanopaper exhibited a good tensile strength of 75.7 MPa at 2.45% strain.

## 1. Introduction

Recent research exploitations on the use of natural fibers as a suitable replacement for petrochemical based composite have revealed the great potential in the performance of cellulose fractions. A very prominent use is of cellulose nanofibers (CNFs) in several applications such as water purification, electromagnetic interference (EMI) shielding, biomedical scaffolds, tissue engineering, sensors, optically transparent functional materials etc [1,2,3]. Their abundance in agricultural waste residue has promoted the utilization of these waste residues as a source of cellulose for the varying technological applications. Apart from wood and non-wood sources, CNFs have been extracted from other cellulose based organism such as algae, tunicates, and bacterial cellulose, but the fact remains that the primary source CNFs is natural plant cell walls [4,5]. The use of this biomass has been pivotal on the unique properties such as their renewability, eco friendliness, abundance, low cost, ease of their fiber extraction, and their interfacial bonding ability in polymer composites. On the other hand, kenaf bast fiber (*Hibiscus cannabinus L*.) is an essential natural fiber with antibacterial properties, excellent air permeability, and high mechanical properties. The plant has high economic potential with ecological advantages making it survive a wide range of weather conditions. This has made its cultivation easy, as in three months of plantation it grows to the mature stage of about 3 m tall and with a base diameter of 3–5 cm. In addition to its excellent economic potential, kenaf fibers have gained considerable attention due to their high cellulose content. [6].

Several techniques have been employed for the extraction of CNFs from the biomass including high-pressure homogenization [7], grinding [8], cryocrushing [9], high-intensity ultrasonication [10], steam explosion process [11], electrospinning [10], high speed blending [12], 2,2,6,6-Tetramethylpiperidin-1-yl)oxyl (TEMPO) oxidation [13,14,15], and extrusion process [16]. Recently the steam explosion technique has proved as an efficient technique to extract CNFs from the biomasses. The CNFs extracted via the steam explosion technique have been used to improve the properties of products, providing strength enhancement in composites, paper manufacturing, and the creation of new products [17,18]. The primary purpose of this research is to extract CNFs via a supercritical carbon dioxide (SC-CO_2_) process. In attempts to develop improved cellulose pretreatment techniques, the idea of using SC-CO_2_ explosions with a low temperature (compared with the steam explosion) and possibly reduced expense (compared with ammonia explosions) as an alternative method [19].

In the SC-CO_2_ process, carbon dioxide (CO_2_) under very high pressure (50 MPa) penetrates the lignocellulosic structures by diffusion. From the literature, the SC-CO_2_ process has been successfully used in oil and fat extraction from oilseed plants such as the seeds of hazel, sunflower, peach, jojoba, and palm [20]. Besides, it has been used in degumming cellulosic fiber and acidification of palm oil [21]. In other words, the SC-CO_2_ process has the potential for the defibrillation of cellulose materials [22].

This is the first report on the production of CNFs via eco-friendly supercritical carbon dioxide (SC-CO_2_) treatment followed by mild oxalic acid hydrolysis, and thereby the fabrication of the cellulose nanopapers from the resultant CNFs suspension. Cellulose nanopapers are a class of promising functional materials that can be explored for diverse applications ranging from gas barrier films, water purification membranes, flexible optoelectronic devices, substrates for sensors, and EMI shields, due to its excellent mechanical properties, thermal stability, high aspect ratio etc. [23].

## 2. Materials and Methods

### 2.1. Materials

Kenaf plant was collected from Nibong Tebal Paper Mills, Seberang Perai, Pulau Pinang, Malaysia. Its bast fibers were separated from its inner core by using a Sprout Bauer refiner (USA) model number R34EM. SC-CO_2_ was procured from ZARM Scientific and Supplies (Malaysia).

### 2.2. Isolation of CNFs via Supercritical Carbon Dioxide Assisted Mild Acid Hydrolysis

Initially, raw kenaf fiber of about 500g (oven-dried weight) was heated in an alkaline solution containing 25 wt % of sodium hydroxide (NaOH) and 0.2 wt % of anthraquinone (AQ) at 160 °C for 3.5 h. After this chemical pulping process, alkali treated fibers were obtained. Then, the alkali treated fiber was washed with distilled water to remove the degraded hemicellulose and extractives. Subsequently, a totally chlorine free (TCF) bleaching treatment (oxygen bleaching, ozone bleaching and hydrogen peroxide bleaching) was performed to eliminate lignin using 3% hydrogen peroxide (H_2_O_2_), 3% sodium hydroxide (NaOH), and 0.5% magnesium sulfate (MgSO_4_) at 80 °C for 2 h. Then the bleached fibers were collected and thoroughly washed to a neutral pH. Next, the bleached fibers were subjected to the SC-CO_2_ explosion with 50 MPa at 60 °C for 2 hours as a pretreatment process. As a result, SC-CO_2_ cellulose nanofibers (SC-CO_2_ CNFs) were obtained. Finally, mild acid hydrolysis was used to avoid toxicity, with low concentration of 5% oxalic acid, and was implemented on the SC-CO_2_ CNFs to produce cellulose nanofibers (CNFs). The UV/Vis transmittance test was done to the CNF suspension of 0.3 wt % in quartz cuvettes using the Shimadzu spectrophotometer UV–Vis 1650pc at 400 nm to 800 nm using distilled water as the reference.

### 2.3. Fabrication of Cellulose Nanopaper Using Extracted CNFs Suspension

0.3 wt % of CNFs suspension was used for the fabrication of the cellulose nanopaper via simple vacuum filtration process. A filtration assembly with cellulose ester membrane (47 mm in diameter and 0.45 µm pore size) was employed for the vacuum filtration of the CNFs suspension. After this, the fabricated cellulose nanopaper was peeled off from the filter and dried for 15 mins in a hot press at 60 °C.

### 2.4. Characterization

Several analytical methods, including transmission electron microscopy TEM analysis, Fourier transform infrared FTIR spectroscopy analysis, thermogravimetry analysis TGA, X-ray diffraction XRD analysis, and the zeta potential test were performed. This was to study the morphology of the cellulose nanofiber, chemical structure, thermal properties, and dimensions of the extracted CNFs.

#### 2.4.1. X-Ray Diffraction (XRD)

X-ray diffraction profiles of the fibers at every treatment were collected by a JEOL diffractometer, Model JDX 8P, using CuKα radiation at the operating voltage and current of 30 kV and 20 mA, respectively. The diffraction intensities were scanned and recorded between 0° and 30° (2θ angle range).

#### 2.4.2. Scanning Electron Microscopy (SEM) Analysis

Scanning electron microscopy (SEM) (EVO MA-10; Carl Zeiss, London, UK) was used to investigate the morphology of the virgin and treated fibers. The tested samples were gold coated with a Fisons Polaron sputter coater (Polaron SC500, London, UK) before the examination.

#### 2.4.3. Transmission Electron Microscopy (TEM) Analysis and Evaluation of Surface Area of the Extracted Cellulose Nanofibers.

TEM of the extracted CNFs were taken with a Philips CM 30 transmission electron microscope with an acceleration voltage of 75 kV. A drop of a diluted suspension (1 wt %) was deposited on the surface of a clean copper grid and coated with a thin carbon film. As for contrast in TEM, the cellulose nanofibers were negatively stained in a 2 wt % solution of uranyl acetate. N_2_ adsorption–desorption experiments were conducted at 77 K using a Micrometrics Surface and Pore Size Analyzer, model ASAP2020. Prior to the experiment the sample was degassed at 473 K for 18 h. The surface area of the extracted cellulose nanofibers was obtained using the Brunauer–Emmett–Teller (BET) model surface area analyser.

#### 2.4.4. Zeta Potential Measurements

The zeta potential measurements of the extracted CNFs were determined using a Malvern Zetasizer Nano ZS 7.11 (Malvern Instruments Ltd., Malvern, UK) by the DLS technique. Distilled water with a refractive index of 1.330 was added to the cell as a dispersant, and then the samples were sonicated for 10 minutes. The measurement was carried out with the set parameters such as viscosity of 0.8872, dispersant and material refractive indexes RI of 1.3330 and 1.47 respectively, while the pH value of each suspension was adjusted by adding either NaOH or HCl. Zeta potential measured as a function of the concentration of original aqueous CNFs suspension in 0.1 mM KCl electrolyte.

#### 2.4.5. Evaluation of the Mechanical Properties of the Fabricated Cellulose Nanopaper

Tensile testing of the fabricated cellulose nanopaper was done using a universal testing machine Tinus Olsen 50 KT coupled with a 100-N load cell. Cellulose nanopaper was cut into a strip of 30 mm in length and 3–5 mm in width. The tensile strength of cellulose nanopapers with varying concentration, 0.1%, 0.2%, and 0.3% were evaluated. The samples were tested with a speed of 1mm/min up to 7% tensile stress. The measurements were taken five times.

## 3. Results and Discussion

### 3.1. Morphology of the Extracted Cellulose Nanofibers

**Figure 1:**
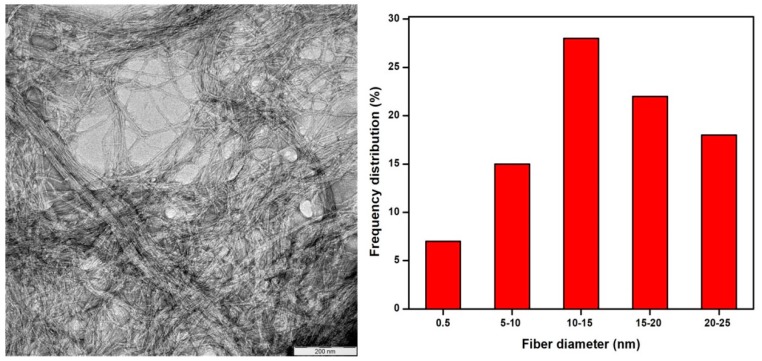
TEM image and diameter frequency distributions of supercritical carbon dioxide (SC-CO_2)_ cellulose nanofibers (CNFs) from kenaf fibers (inset scale is 200 nm).

Figure 1 illustrates the TEM image and diameter frequency distributions of CNFs from kenaf fibers. From Figure 1, it can be concluded that the extracted CNFs were in the range between 10–15 nm. In order to measure the diameters of the cellulose nanofibers, Image J software was used. From Figure 1, it is shown that the SC-CO_2_ assisted mild acid hydrolysis is an efficient technique to extract CNFs from the kenaf fibers. It was expected that, during the SC-CO_2_ technique, the lignocellulosic structure undergoes chemical decomposition [24,25]. Moreover, the extracted CNFs had a BET surface area of 10.362 m2/g. Cellulose materials extracted from plants are often surrounded by a plethora of lignin and hemicellulose fibers which makes them less susceptible to the acid hydrolysis. Thus, they are subjected to many pretreatments, such as SC-CO_2_ explosions that are non-toxic and economically viable.

Furthermore, the high pressure applied could also disrupt the secondary interactions among the fibers, thereby defibrillating the materials and increasing the surface area of the substrate for subsequent mild oxalic acid hydrolysis [19,26,27,28].

### 3.2. FT-IR Studies

**Figure 2:**
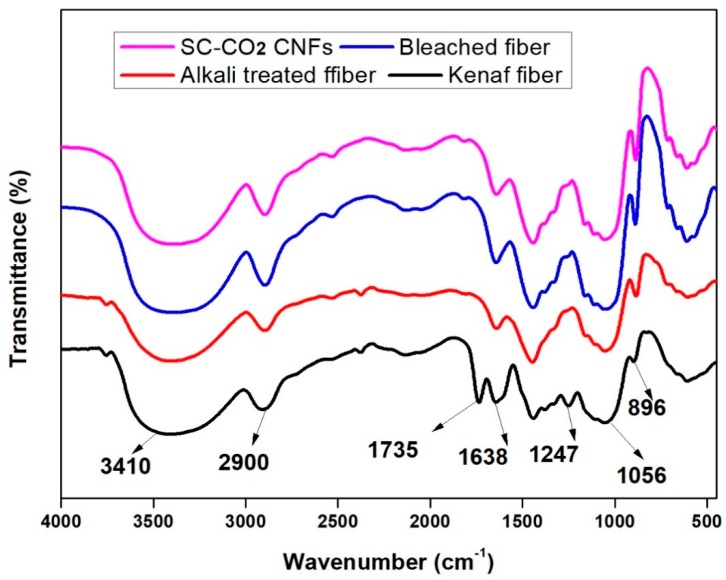
Showing the Fourier transmission infra-red (FTIR) of (**a**) raw kenaf fiber, (**b**) alkaline treated kenaf fiber, (**c**) bleached kenaf fiber, and (**d**) SC-CO_2_ extracted CNFs.

The effect of the various treatments on the functional groups present in the kenaf fibers was monitored with the FT-IR spectra, as shown in Figure 2.

**Figure 3:**
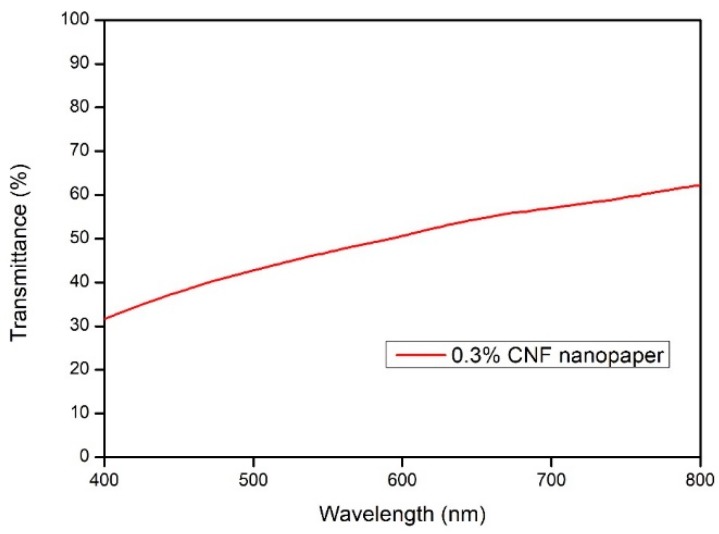
The UV/Vis trasmittance of 0.3 wt % CNF nanopaper.

The appearance, disappearance and the alteration of the spectra band sizes at the fingerprint portion indicate the effectiveness of the treatment. The appearance of identical FTIR spectra at 3410 cm^−1^ and 1638 cm^-1^ (which indicates aromatic OH stretching) and the spectra band 2900 cm^−1^ (which signifies CH stretching vibration) indicates that the various treatments carried out on the fibers had not influenced the chemical structure of the treated fiber [29]. This shows that the chemical components of the fibers did not change all through the isolation of cellulose nanofiber through alkali hydrolyzed fiber degumming assisted SC-CO_2_. However the small peak observed at 1735 cm^−1^ in the raw kenaf fiber is ascribed to the acetyl and uronic ester groups of hemicellulose or the ester linkage of carboxylic group of ferulic and ρ-coumaric acids of lignin and/or hemicelluloses, which disappeared in the treated fibers indicating removal of the hemicelluloses and lignin as the fiber treatment progressed [30]. The absorption peak at 1247 cm^-1^ also disappeared, as can be seen in the raw fiber spectra that is presenting the C-O out of plane stretching vibration of the lignin aryl group. These two findings prove that most of the hemicellulose and lignin had become utterly absent from the spectra by the chemically treated kenaf bast fibers. The peak detected in all the spectra at 1056 cm^-1^ is due to the C-O-C pyranose ring skeletal vibration. The increasing intensity of this band showed a rise in fibers crystallinity and the cellulose content. The spectra peak intensities around 896 cm^−1^, which increase with the fiber treatment, can be attributed to the glycosidic linkages and glucose units of the cellulose [31]. The gradual increase in the spectra band from the raw sample to the SC-CO_2_ extracted CNFs indicate the removal of hemicellulose and lignin portions step by step during each treatment. The UV transmittance test was done on the fabricated CNF nanopaper (Figure 3) in the range of 400 to 800 nm. The results showed that, the higher the transmittance percentage, the transparency will increase, and more fibrillation will occur on the fibers [32,33].

### 3.3. X-Ray Diffraction XRD Analysis

**Figure 4:**
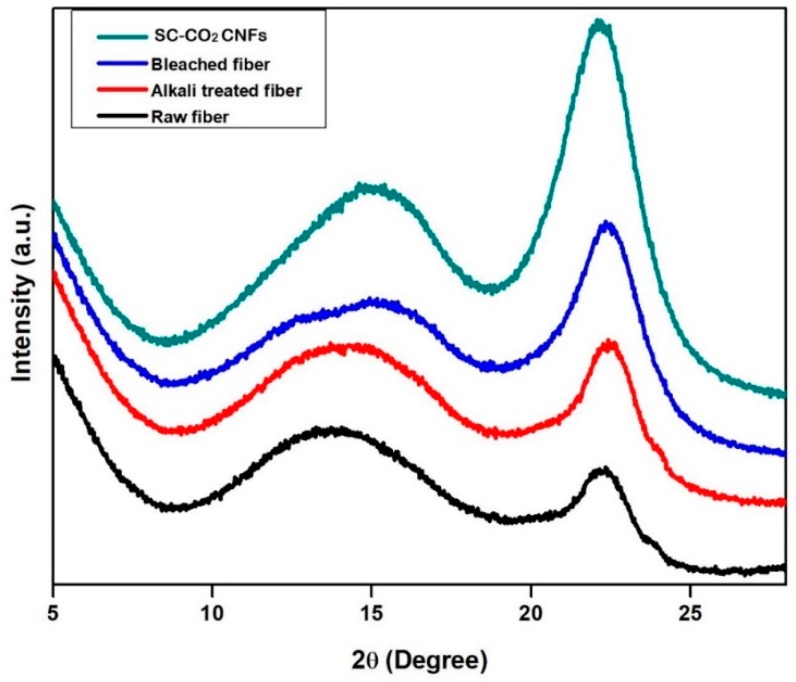
XRD of raw kenaf fiber, alkali treated kenaf fiber, bleached kenaf fiber, and SC-CO_2_ extracted CNFs.

Figure 4 shows the XRD diffraction profiles of raw fiber, alkali treated fiber, bleached fiber, and SC-CO_2_ extracted CNFs. The crystallinity of the CNFs is a crucial factor for determining the thermal and mechanical properties. The inter hydrogen bonding between the hydroxyl groups of CNFs results in the perfect crystalline packing, which significantly influences the mechanical properties.

From the Figure 4, the crystallinity index of the fibers was gradually increasing after each treatment. The intense peak in the SC-CO_2_ extracted CNFs indicates the efficient and complete removal of amorphous portions like lignin and hemicellulose during the SC-CO_2_ assisted technique coupled with mild acid hydrolysis. All samples showed similar diffraction profiles and extracted CNFs show diffraction peaks around 2θ = 16.3° and 2θ = 22.6°, which typically represent cellulose type I [24,25]. Generally, cellulose found in the plant sources is in cellulose type I nature. The crystallinity indices (CrI) of all samples were evaluated by using the method of Segal, as given in Equation (1) [31].

CrI = [(*I*_200_ − *I*_AM_)/*I*_200_] × 100 (1)

In Equation (1), CrI expresses the relative degree of crystallinity, *I*_200_ is the maximum intensities of the 200 lattice diffraction at 2θ = 23°, and *I*_AM_ is the intensity of diffraction at 2θ = 18°. *I*_200_ represents both the crystalline and amorphous regions, while *I*_AM_ represents only the amorphous portion. The crystallinity index of the SC-CO_2_ extracted CNFs was 92.8%, whereas raw fiber, alkali treated fiber, and bleached fiber were 33.2%, 54.6%, and 88.4%, respectively.

### 3.4. Stability of the Extracted CNFs Suspension via SC-CO_2_ Assisted Mild Acid Hydrolysis

**Figure 5:**
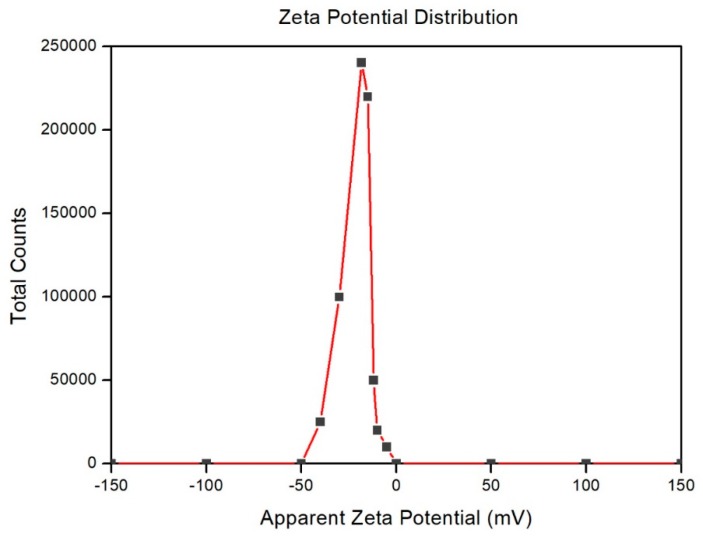
Zeta potential value of the SC-CO_2_ extracted CNFs.

Zeta potential (estimated as surface charge) can be measured by tracking the rising rate of negatively or positively charged particles across an electric field. Figure 5 shows the zeta potential value of the extracted CNFs suspension via SC-CO_2_ assisted mild acid hydrolysis. From Figure 5, the extracted CNFs suspension shows the high negative value of –31.0 ± 4.21 mV, which was due to the SC-CO_2_ treatment coupled with mild oxalic acid hydrolysis. This high negative zeta potential of the extracted CNFs was due to the presence of oxalate groups which were formed during mild oxalic acid hydrolysis. Generally, cellulosic surfaces exhibit a bipolar character with prevalent acidic contribution due to the proton of the hydroxyl functional group as well as of present carboxyl groups from oxalic acid [34].

From the value of the zeta potential, it can be concluded that the suspension of CNFs is stable because the absolute value was higher than –25 mV [34]. This value signifies that there was enough mutual repulsion resulting in colloidal stability which could be an essential requirement for improving the mechanical properties of the nanocellulose based composites [35].

### 3.5. Thermogravimetric Analysis (TGA)

**Figure 6:**
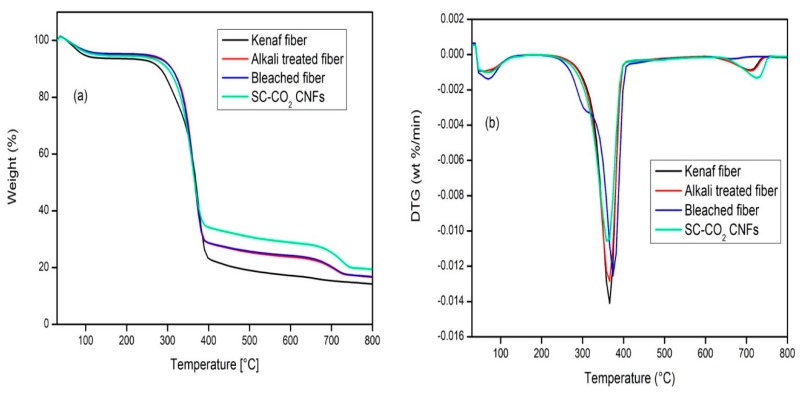
(**a**) TGA and (**b**) DTG curves of raw kenaf fiber, alkali treated fiber, bleached fiber, and SC-CO_2_ extracted CNFs.

The thermogravimetric analysis (TGA) and Derivative Thermogravimetric (DTG) curves of raw kenaf fiber, alkali treated fiber, bleached fiber, and SC-CO_2_ extracted CNFs were shown in Figure 6.

The chemical composition, structure and degree of crystallinity of the lignocellulosic materials contribute majorly to their thermal behavior. From Figure 6(a,b) the thermal degradation of both the raw and SC-CO_2_ extracted CNFs showed three degradation steps. The first degradation started in the range of 50–120 °C for all the samples which gave an initial weight loss due to the evaporation of loosely bound moisture on the surface of the fibers [36]. The second stage, with temperatures in between the 250 to 400 °C range, was caused by the degradation of hemicellulose and the depolymerization of the glycosidic linkage of cellulose. Then, from 600 °C, there followed a gradual lignin degradation coupled with char formation [37]. From the TGA curves, raw kenaf bast fiber has the least thermal stability while the SC-CO_2_ extracted CNFs has the highest thermal stability.

The thermal stability increases with the increase in the fiber treatment, with (in terms of thermal stability) the alkaline treated kenaf fiber, followed by the raw fiber, followed by the bleached kenaf fiber, while the SC-CO_2_ extracted CNFs has the highest thermal stability. This result could be attributed to the gradual removal of amorphous parts of the raw kenaf fiber such as pectin, hemicelluloses, etc, which decomposes before lignin and cellulose as a result of the presence of the acetyl groups in hemicellulose molecules as well as a higher degree of crystallinity in the chemically treated fiber samples. Hemicellulose has a random amorphous structure, and it could be easily hydrolyzed. Another amorphous substance is lignin which is different from hemicellulose in that it is composed of three kinds of benzene-propane units, being heavily cross-linked and having a very high molecular weight. The thermal stability of lignin is thus very high, and it is difficult to decompose quickly [38]. Thus, the higher crystallinity of the extracted fiber led to higher thermal degradation. Besides that, the cellulose molecule is a very long polymer of glucose units, and its crystalline regions could improve the thermal stability of lignocellulosic fibers [39]. It was observed that SC-CO_2_ extracted CNFs had the highest residual weight (19.5%) at 800 °C, followed by bleached fiber (16.9%) and then alkali-treated (16.7%), and with the lowest residual weight for raw Kenaf fiber (14.4%). Because of the above results, it can be concluded that the SC-CO_2_ extracted CNFs have excellent thermal stability and will be suitable in the production of green biocomposites.

### 3.6. Morphology of the Treated and Untreated Fibers

**Figure 7:**
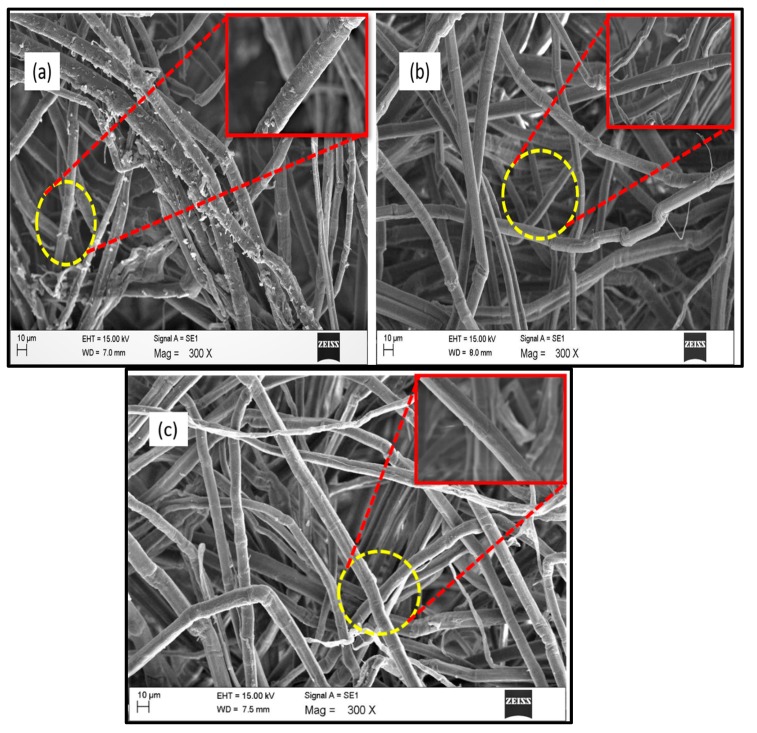
SEM images of (**a**) raw fiber, (**b**) alkali treated fiber, and (**c**) bleached fiber.

SEM images of the kenaf fiber at different processing stages are shown in Figure 7. From Figure 7(a) the raw fiber exhibited a smoother surface. Figure 7(b) shows the SEM image of the alkali treated fiber which exhibited a rougher surface compared with the raw fiber, due to the partial removal of amorphous portions like lignin, hemicellulose, pectin, wax, and other impurities during the alkali treatment. After alkali treatment, there is little difference in the diameter of fiber bundles due to the presence of lignin. Lignin forms a bridge bond with the cellulose and acts as a binding material in fiber components and thus maintaining the bundle form even after alkali treatment [40]. The morphology of the bleached kenaf fiber is shown in Figure 7(c), From the Figure 7(c)**,** it is shown that the fiber bundle becomes defibrillated which resulted in the reduction of the fiber diameter after bleaching treatment. This reduction in fiber diameter during bleaching was due to the successive removal of lignin (binding material) by bleaching treatment, which facilitates fibers separated into an individual form. The deterioration of natural fiber bundles during bleaching was reported by Hornsby et al., and they found that the reduction of the fiber diameter of the natural fiber bundles after bleaching treatment was due to the separation of the primary cell wall of the natural fiber due to the removal of the amorphous or cementing materials like lignin and hemicellulose [41].

### 3.7. Strategy for the Fabrication of Cellulose Nanopaper

**Figure 8:**
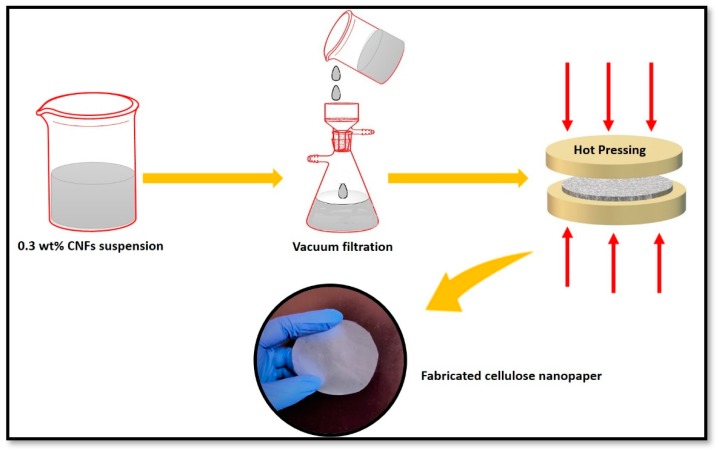
Diagram depicting the fabrication of cellulose nanopaper.

Figure 8 depicts the fabrication of cellulose nanopaper via a vacuum filtration technique. CNFs suspension (at 0.3 wt %) was used for the fabrication of the cellulose nanopaper. A filtration assembly with cellulose ester membrane (47 mm in diameter and 0.45 µm pore size) was employed for the vacuum filtration of the CNFs suspension. After this, the fabricated cellulose nanopaper was peeled off from the filter and dried for 15 mins in a hot press.

### 3.8. Morphology of the Fabricated Cellulose Nanopaper

**Figure 9:**
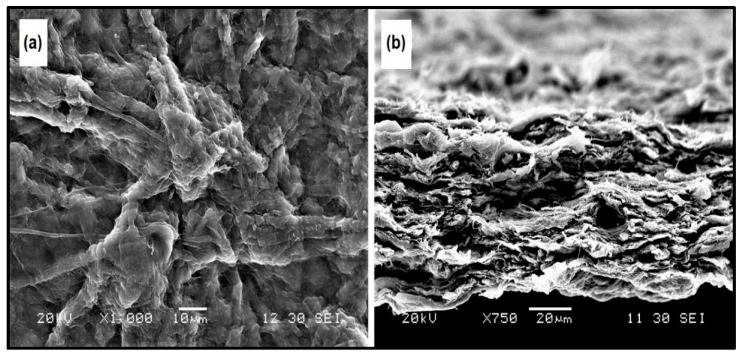
SEM image of (**a**) surface of cellulose nanopaper, and (**b**) cross-section of cellulose nanopaper.

The cellulose nanopapers were fabricated via a simple vacuum filtration process of the 0.3 wt % of CNFs suspension. Figure 9(a) shows the SEM image of the surface of fabricated cellulose nanopaper and it is clearly shown that the CNFs were fused together. During the vacuum filtration process, individual CNFs fibers fused together to form larger bundles of fibers due to the strong intermolecular hydrogen bonding [23].

Figure 9(b) shows the cross-section of the fractured cellulose nanopaper, and it was clearly shown that cross section of cellulose nanopaper displayed a layered structure due to the intermolecular hydrogen bonding between the CNFs [23].

### 3.9. Mechanical Strength of the Fabricated Cellulose Nanopaper

**Figure 10:**
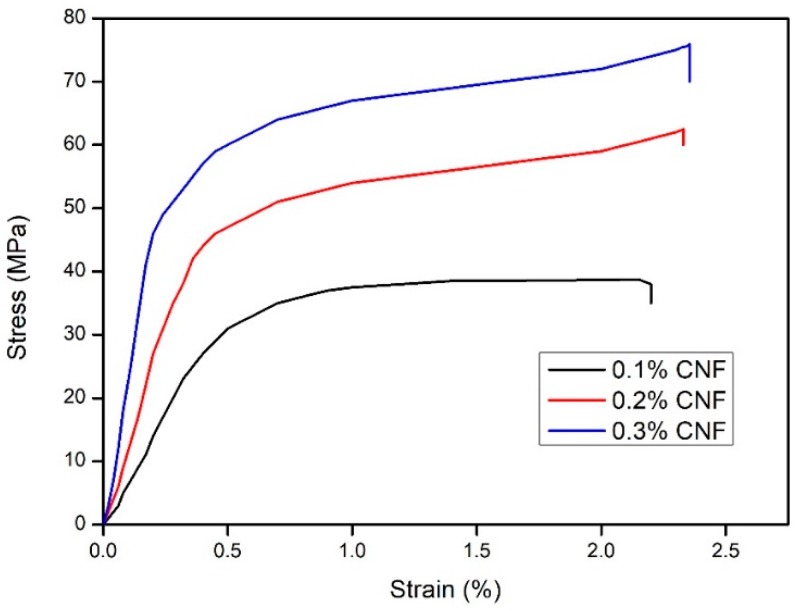
Tensile stress vs. strain curves of the fabricated cellulose nanopapers with varying concentrations.

The mechanical properties of the cellulose nanopapers are very relevant for varying technological applications. Figure 10 shows the tensile stress versus strain curves for neat cellulose nanopaper. From Figure 10, it was shown, at 2.45% of strain, the fabricated cellulose nanopaper showed a tensile strength of 75.7 MPa. This result was comparable with Sehaqui et al., where they fabricated cellulose nanopaper via a liquid CO_2_ evaporation (L-CO_2_) technique, and the fabricated cellulose nanopaper had a tensile strength of 20 MPa [42]. From the Figure 10, it was found that, the highest tensile strength was obtained for 0.3% of CNF, whereas 0.1% CNF and 0.2% CNF had tensile strengths of 37 MPa and 58.6 MPa, respectively. The intermolecular hydrogen bonding between the hydroxyl groups of CNFs in the cellulose nanopaper resulted in the excellent mechanical strength. Additionally, the tightly packed nanofibrillar network and numerous fiber-fiber hydrogen bonds could also result in the compact nature of the cellulose nanopaper [23]. Table 1 illustrates the tensile strength of the cellulose nanopapers fabricated via various techniques, and the mechanical properties of CNF nanopapers with different CNF content are shown in Table 2.

## 4. Conclusions

CNFs were successfully prepared via SC-CO_2_ treatment followed by mild acid hydrolysis of kenaf TCF bleached fiber. During the alkali and bleaching treatment, the amorphous portions like hemicellulose and lignin were removed respectively, as shown in the FTIR studies. TEM images revealed that extracted CNFs have a diameter in the range of 10–15 nm and a length of up to several micrometers. From TEM images, it can be concluded that SC-CO_2_ treatment followed by mild acid hydrolysis is an efficient technique to extract CNFs from the plant biomass. XRD diffraction profiles of the extracted CNFs show typical cellulose I structures. The zeta potential of extracted CNFs show the high negative value of –31.0 ± 4.21 mV which indicates the stable dispersion of CNFs due to the stabilization of the surface anionic charges of the CNFs. Additionally, cellulose nanopapers were fabricated via a simple vacuum filtration technique using the CNFs extracted via SC-CO_2_ treatment followed by mild acid hydrolysis. Mechanical studies proved that the CNFs yielded cellulose nanopaper with a tensile strength of 75.7 MPa at 2.45% strain. The fabricated cellulose nanopaper could be a promising substrate for varying technological applications. We firmly believe that the demonstrated SC-CO_2_ treatment followed by mild acid hydrolysis technique will be an effective platform to design eco-friendly techniques for the extraction of CNFs from plant biomass in the nearby future.

## Figures and Tables

**Table 1:** Tensile strength of some cellulose nanopapers fabricated via various techniques.

Sample Name	Tensile Strength (MPa)	References
CNF nanopaper from Kenaf fiber	75.7	This work
Cellulose powder (KC Flock, W-50) after 4 passes through high pressure homogenizer	71.3	[43]
Nanofibrils from wood pulp fibers	129.0	[44]
Kraftpulp through a high shear stone grinder (Masuko supermasscolloider)	168.0	[45]
Clay nanopaper	124.0	[46]
Enzymatic Cellulose Nanofibrils (NFC) nanopaper from Softwood sulphite pulp fibers	25.0	[42]

**Table 2:** Mechanical properties of CNF nanopapers with different CNF content.

CNF Content	Density (m3/kg)	Bulk (kg/m3)	Tensile Strength (MPa)	Tensile Modulus (GPa)	Elongation at Break (%)
0.1% CNF	560 ± 2.1	1786	37.0	845.9	16.092
0.2% CNF	574 ± 1.5	1742	58.6	1728.1	18.664
0.3% CNF	590 ± 1.9	1695	75.7	1885.8	22.744
